# Diagnostic and prognostic value of serum periostin in patients with non-small cell lung cancer

**DOI:** 10.18632/oncotarget.13004

**Published:** 2016-11-01

**Authors:** Chun-Hua Xu, Wei Wang, Yong Lin, Li-Hua Qian, Xiu-Wei Zhang, Qing-Bo Wang, Li-Ke Yu

**Affiliations:** ^1^ Endoscopic Center of Nanjing Chest Hospital, Nanjing, Jiangsu 210029, China; ^2^ Clinical Center of Nanjing Respiratory Diseases and Imaging, Nanjing, Jiangsu 210029, China; ^3^ Department of Respiratory Medicine, Nanjing Chest Hospital, Nanjing, Jiangsu 210029, China; ^4^ Department of Respiratory Medicine, Nanjing Pukou Central Hospital, Nanjing, Jiangsu 211800, China; ^5^ Department of Respiratory Medicine, Affiliated Jiangning Hospital of Nanjing Medical University, Nanjing, Jiangsu 211100, China; ^6^ Department of Geriatrics Medicine, Nanjing Second Hospital, Nanjing, Jiangsu 210003, China

**Keywords:** periostin, non-small cell lung cancer, prognosis, diagnosis, biomarker

## Abstract

The periostin protein is expressed in a variety of human malignancies. The aim of this study was to explore the diagnostic and prognostic value of serum periostin levels in patients with non-small cell lung cancer (NSCLC). We measured serum periostin levels by ELISA in 296 NSCLC patients, 120 benign lung diseases (BLD) patients and 160 healthy controls. The levels of serum periostin in NSCLC patients were significantly elevated compared with those in healthy controls (*P* < 0.001) and BLD patients (*P* < 0.001). Using a cutoff value of 30.87 ng/ml, the sensitivity and specificity of periostin in differentiating between NSCLC patients and BLD patients, and between NSCLC patients and healthy controls was, 48.6 and 91.7%, and 51.4 and 97.5%, respectively. Kaplan-Meier log rank analysis revealed that the higher serum periostin levels group had a poorer progression-free survival (PFS) and overall survival (OS) compared with lower periostin group (*P* = 0.024, *P* = 0.015, respectively). Further univariate and multivariate Cox regression analysis showed that serum periostin was an independent risk factor of prognosis of NSCLC patients. In conclusion, our study suggests that serum periostin could be considered as a diagnostic and prognostic marker for NSCLC patients.

## INTRODUCTION

Lung cancer is the leading cause of cancer-related death in the world [1]. Non-small cell lung cancer (NSCLC) accounts for up to 80–85% of lung cancer [2]. When first diagnosed, most of the lung cancer patients have advanced stage, so that patients have little effective treatment, and this presents five years survival rates of less than 15% [3, 4]. Several tumor markers such as carcinoembryonic antigen (CEA), cytokeratin 19 fragment (CYFRA21-1), and neuron-specific enolase (NSE) have been used as biomarker for lung cancer. Nevertheless, none of them showed satisfactory for diagnosis because of their low sensitivity and specificity. Hence, biomarkers for diagnosis and prognosis of lung cancer are urgently needed [5].

Periostin, a secreted matrix N-glycoprotein, which is a component of NH2-terminal signal peptide sequence, internal homologous repeats, cysteine-rich domain, and hydrophilic COOH-terminal domain [6, 7]. Previous studies showed that periostin was overexpressed in various human tumors [8–11]. Many studies have indicated that periostin overexpression or raised serum periostin level is associated with poor patient outcomes [12, 13]. Periostin is overexpressed in lung cancer tissues and associated with the prognosis [14–16], but the correlation between serum periostin and NSCLC progression, as well as the effects of periostin on survival of NSCLC, has not been fully assessed.

In the present study, we explored the correlation between serum periostin and clinicopathological variables and patient survival. Our results showed that the evaluation of serum periostin could be a valuable biomarker for NSCLC.

## RESULTS

### Clinical characteristics

The mean age of the NSCLC patients (56.5 ± 12.7 years) was not obviously different from BLD patients (55.8 ± 11.8 years) and healthy controls (57.8 ± 11.7 years). The proportion of male gender is 60.8% of the NSCLC patients, 58.3% of the BLD patients and 60.0% of the healthy controls, respectively, with no significantly difference (*P* > 0.05). At the time of diagnosis, 110 patients were at stage I + II and 186 patients were at stage III + IV, 41.9% of the patients with lymph node metastases, and 56.4% with distant metastases. The patients’ clinical characteristics are presented in Table 1.

**Table 1:** The characteristics of NSCLC patients, BLD patients and healthy controls

Variables	NSCLC	BLD	Healthy controls	*P* value
Subject, No	296	120	160	
Age (year)	56.5 ± 12.7	55.8 ± 11.8	57.8 ± 11.7	> 0.05
Gender (n,%)				> 0.05
Male	180 (60.8)	70 (58.3)	96 (60.0)	
Female	116 (39.2)	50 (41.7)	64 (40.0)	
Smoking condition				> 0.05
Non-smoker	160 (54.1)	65 (54.2)	90 (56.3)	
Smoker	136 (45.9)	55 (45.8)	70 (43.7)	
Histology				
ADC	164 (55.4)			
SCC	132 (44.6)			
Lymph node metastasis				
Negative	172 (58.1)			
Positive	124 (41.9)			
Distant metastases				
Negative	129 (43.6)			
Positive	167 (56.4)			
Stage				
I-II	110 (37.2)			
III-IV	186 (62.8)			
BLD				
Tuberculosis		40 (33.3)		
Bronchiectasis		30 (25.0)		
Lung bullae		30 (25.0)		
Inflammatory pseudotumor		20 (16.7)		

Abbreviations: NSCLC, non-small-cell lung cancer; BLD, benign lung diseases; ADC, Adenocarcinoma; SCC, Squamous cell carcinoma.

### Serum levels of periostin in NSCLC patients, BLD patients and healthy controls

**Figure 1:**
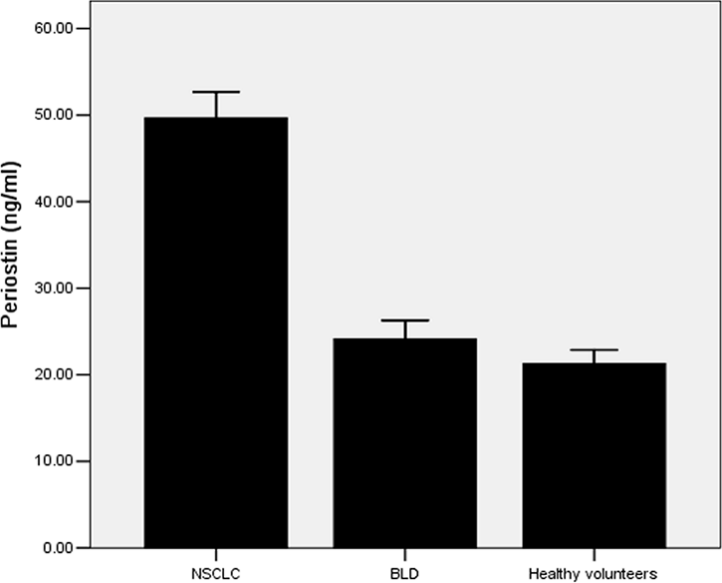
Serum levels of periostin in NSCLC patients, BLD patients and healthy controls Among 296 NSCLC patients, the serum levels of periostin were (49.64 ± 8.46) ng/ml, which were significantly higher than those of BLD patients (24.11 ± 4.67) ng/ml and healthy controls (21.27 ± 3.42) ng/ml (*P* < 0.001)

As shown in Figure 1, the levels of serum periostin were significantly higher in NSCLC patients compared with those in healthy controls (49.46 ± 8.46ng/ml vs. 21.27 ± 3.42ng/ml, *P* < 0.001), and those in BLD patients (49.46 ± 8.46ng/ml vs. 24.11 ± 4.67ng/ml, *P* < 0.001). However, Serum periostin levels were similar in healthy controls and BLD patients (*P* > 0.05). These results show that periostin can be used as a biomarker for the diagnosis of lung cancer.

### ROC analysis of serum periostin levels in NSCLC patients

**Figure 2:**
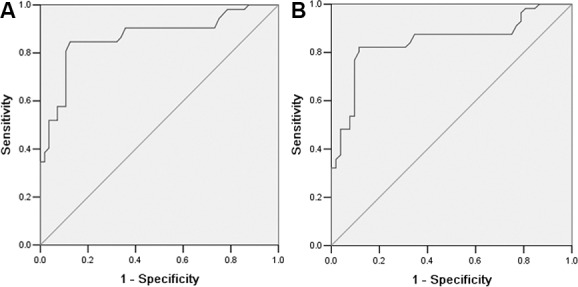
ROC analysis of periostin for differentiation of NSCLC patients from healthy controls (A) and from BLD patients (B) The analysis resulted in an AUC of 0.867 (NSCLC patients vs. healthy controls) and 0.846 (NSCLC patients vs. BLD patients), respectively.

To evaluate the value of serum periostin as a marker, ROC curves was applied to calculate the sensitivity and specificity of this marker in separating NSCLC patients from healthy controls and BLD patients. As shown in Figure 2, an area under the curve (AUC) value for serum periostin reached 0.867 and 0.846, respectively. Using a cutoff value of 30.87 ng/ml, the sensitivity and specificity of serum periostin in differentiating between NSCLC patients and BLD patients, and between NSCLC patients and healthy controls was, 48.6 and 91.7%, and 51.4 and 97.5%, respectively. These results showed that periostin was a worthy diagnostic marker for NSCLC.

The diagnostic value of periostin in combination with CEA was also analyzed. The results indicated that the sensitivity and specificity of these two markers in differentiating between NSCLC patients and BLD patients, and between NSCLC patients and healthy controls was, 61.8 and 95.0%, and 72.3 and 98.8%, respectively.

### Association between periostin levels and clinicopathological variables

The association between the periostin levels and the clinicopathologic variables in NSCLC patients was analyzed, and the result is summarized in Table 2. The levels of periostin were higher in III-IV stage NSCLC patients compared with those in I-II stage patients (*P* = 0.008). Furthermore, the serum levels of periostin were obviously higher in patients with lymph node metastases than those without (*P* = 0.012). Meanwhile, statistically significant differences in periostin levels were found between NSCLC patients with distant metastases and those patients without distant metastases (*P* = 0.001). However, periostin levels were not associated with age (*P* = 0.714), gender (*P* = 0.412), histology (*P* = 0.367), and smoking condition (*P* = 0.923). After all, these results indicated that periostin levels in NSCLC patients were associated with the progression and metastasis, which could be considered as a potential biomarker to predict NSCLC patients.

**Table 2:** Serum levels of periostin in the three groups and clinical features in the NSCLC group

Group	*N*	Periostin (ng/ml)	*P* value
Healthy controls	160	21.27 ± 3.42	
BLD	120	24.11 ± 4.67	0.001*
NSCLC	296	49.64 ± 8.46	
Clinical variables in NSCLC group			
Age (year)			0.714
≤ 60	100	49.34 ± 8.67	
> 60	196	50.71 ± 8.44	
Gender			0.412
Male	180	49.18 ± 8.02	
Female	116	50.53 ± 9.59	
Smoking condition			0.923
Non-smoker	160	49.47 ± 7.85	
Smoker	136	49.68 ± 9.07	
Histology			0.367
ADC	164	48.78 ± 8.48	
SCC	132	51.88 ± 8.49	
Lymph node metastasis			0.012*
Negative	172	47.02 ± 8.61	
Positive	124	52.83 ± 8.44	
Distant metastases			0.001*
Negative	129	42.67 ± 7.17	
Positive	167	55.39 ± 10.86	
Stage			0.008*
I-II	110	45.81 ± 7.16	
III-IV	186	53.48 ± 8.08	

Abbreviations: NSCLC, non-small-cell lung cancer; BLD, benign lung diseases; ADC, Adenocarcinoma; SCC, Squamous cell carcinoma.

*Significant difference.

### Serum periostin levels indicates a favorable prognosis of NSCLC patients

**Figure 3:**
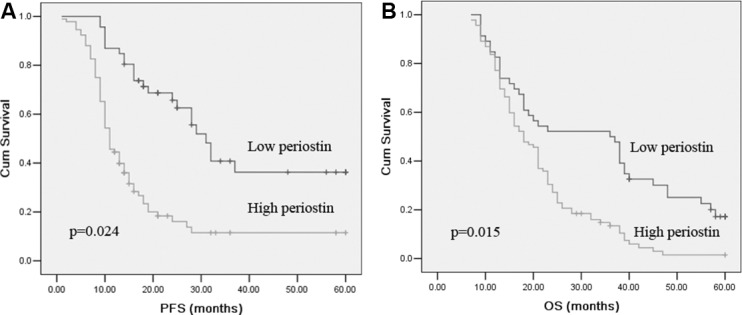
Kaplan–Meier survival curves for PFS (A) and OS (B) in NSCLC patients with high- and low- serum periostin levels group Log-rank test determined that the PFS and OS in high serum periostin group were significantly shorter than those in the low serum periostin group (*P* = 0.024; *P* = 0.015).

To assess the prognostic value of the serum periostin levels, we used serum periostin cutoff value 30.87 ng/ml, which was calculated from ROC analysis, as a threshold to classified NSCLC patients into two groups, high serum periostin group (periostin ≥ 30.87 ng/ml) and low serum periostin group (periostin < 30.87 ng/ml). As shown by Kaplan-Meier log rank analysis, the higher serum periostin levels group was correlated with a shorter PFS and OS, compared with lower serum periostin levels group (*P* = 0.024 and *P* = 0.015, respectively) (Figure 3). Further analysis using univariate and multivariate Cox regression showed that serum periostin levels were independent risk factors of prognosis of NSCLC patients (Table 3). Meanwhile, TNM stage and distant metastases were found to be associated with survival.

**Table 3:** Univariate and multivariate Cox proportional hazards model for OS and PFS in NSCLC patients

Variables	Univariate			Multivariate		
	HR	95 % CI	*P* value	HR	95 % CI	*P* value
OS						
Age (< 60 *vs*. ≥ 60)	0.414	0.057–3.020	0.384	0.999	0.880–1.134	0.983
Gender(female vs. male)	0.812	0.156–4.215	0.804	1.125	0.238–5.318	0.882
Smoking status (smoker *vs*. nonsmoker)	1.245	0.420–3.695	0.692	0.809	0.533–1.227	0.318
Histology(Adenocarcinoma *vs*. Squamous)	0.546	0.249–1.199	0.132	0.971	0.402–2.346	0.949
Lymph node metastasis (Positive *vs*. Negative)	1.395	0.934–2.085	0.104	1.609	0.695–3.723	0.266
Stage (I-II *vs*. III-IV)	0.545	0.312–0.952	0.033*	0.395	0.172–0.909	0.029*
Distant metastases (Positive *vs*. Negative)	1.542	0.672–2.194	0.001*	1.227	1.033–1.458	0.002*
Periostin (high *vs*. low)	1.987	1.025–3.014	0.039*	1.860	1.102–3.918	0.032*
PFS						
Age (< 60 *vs*. ≥ 60)	1.109	0.724–1.698	0.635	0.995	0.963–1.029	0.778
Gender(female *vs*. male)	0.387	0.064–2.360	0.303	0.974	0.229–4.142	0.972
Smoking status (smoker *vs*. nonsmoker)	1.436	0.364–5.663	0.605	0.538	0.132–2.184	0.386
Histology(Adenocarcinoma *vs*. Squamous)	0.664	0.289–1.528	0.336	1.216	0.581–2.544	0.603
Lymph node metastasis (Positive *vs*. Negative)	1.054	0.354–3.138	0.925	1.108	0.684–1.794	0.678
Stage (I-II *vs*. III-IV)	2.342	1.036–5.296	0.041*	2.067	1.003–4.262	0.049*
Distant metastases (Positive *vs*. Negative)	1.352	0.578–1.894	0.001*	1.324	0.678–2.324	0.006*
Periostin (high *vs*. low)	2.152	1.163–3.605	0.015*	2.018	1.123–3.285	0.022*

Abbreviations: CI, confidence interval; DFS, disease free survival; HR, Hazard ratio; OS, overall survival.

*Significant difference.

## DISCUSSION

Periostin is overexpressed in various types of cancer tissues and may affect some aspects of biology, such as tumor angiogenesis, invasion and metastasis [17, 18]. Previous studies have showed that periostin has been upregulated in NSCLC tissue, however, these studies were restricted to quantitative polymerase chain reaction or immunohistochemical assessment of periostin expression and did not concern its serum levels [14, 15, 19]. In the present study, we found that serum periostin levels were increased in NSCLC patients compared with BLD patients or healthy controls. Using a cutoff value of 30.87 ng/ml, serum periostin showed a valuable biomarker for separating NSCLC patients from BLD patients and the individuals. These data reinforced the diagnostic value of periostin levels in NSCLC. The diagnostic value of periostin in combination with CEA was also analyzed. The results indicated that combined detection of these two markers had a better diagnostic value than periostin or CEA alone. This may offer a new method in differentiating NSCLC patients and the controls.

Periostin has been measured in the serum of NSCLC patients by chemiluminescence assays. Notably, this study showed no difference between NSCLC patients and the controls, and there was also no correlation between the periostin level and sex, stage, lymph node status or distant metastasis [20]. However, our results showed that elevated serum periostin levels were associated with advanced disease stage, lymph node and distant metastases, and there was no significant associations of periostin levels with age, gender, smoking condition and histology. These results indicated that periostin levels were associated with the progression and metastasis NSCLC, which could be serve as a potential biomarker to predict NSCLC patients.

In the present work, we observed that there was a significant correlation between serum periostin levels and NSCLC patients survival. Our results indicated that high periostin was significantly associated with a decreased PFS and OS in univariate analysis. This relationship was further demonstrated in the survival curves. Multivariate analysis also showed that periostin was an independent prognostic factor for NSCLC, which is in line with the data reported for tissue periostin expression using immunohistochemical techniques [14, 19]. The prognostic utility of tissue periostin expression has also been established in other malignancies [21, 22]. Concerning the circulating periostin levels, there was strong evidence that serum level of periostin in NSCLC and colorectal cancer patients is significantly elevated [20, 23].

In conclusion, increased serum periostin levels were significantly associated with poor prognosis. Our study provides evidence that periostin can be considered of diagnostic and prognostic utility in NSCLC.

## MATERIALS AND METHODS

### Patients

Three separate groups were included. The first group included 296 patients with NSCLC, who examined at the Nanjing Chest Hospital from January 2011 to May 2014. All patients’ histopathological classification was determined according to the WHO criteria, and staged classification was defined according to the 7th edition of UICC TNM staging system [24]. Follow-up lasted through December 2015, with a median follow-up period of 24 months for living patients (range, 3–60 months). Progression-free survival (PFS) was defined as the time interval between the date of diagnosis and the date of disease relapse. Overall survival (OS) was defined as the time interval between the date of diagnosis and the date of death. The second group enrolled 120 sex- and age-matched consecutive cases with BLD patients. Diagnoses were pulmonary tuberculosis (40 cases), bronchiectasis (30 cases), lung bullae (30 cases) and inflammatory pseudotumor (20 cases). The third patient group included 160 sex- and age-matched healthy volunteers. Serum samples from this subject group were offered from Central Laboratory of Nanjing Chest Hospital.

The study protocol was approved by the ethics committee of Nanjing Chest Hospital. All patients provided written informed consent before enrollment.

### Measurement of serum periostin and CEA levels

Serum samples from each individual were obtained at the time of diagnosis, before any therapeutic measures were started. Samples were centrifuged at 1500 × g for 10 min at −4°C. The supernatant was stored at −80°C. The periostin levels were determined by ELISA with the commercial periostin ELISA Ready-SET-Go kit (eBioscience, San Diego, CA). The levels of CEA were measured by electrochemiluminescence immunoassays on Roche Elecsys 1010 analyzer (Roche Diagnostics; Mannheim, Germany). The upper normal limit for the CEA is 5 ng/ml. All samples were blinded to the technologists running the assays, and the code was broken to the statisticians after the database was constructed.

### Statistical analysis

Statistical software (SPSS for Windows, version 18) was used for the analysis. Differences between independent groups were examined by the Mann-Whitney U test. To determine the diagnostic accuracy of periostin, receiver operating characteristic (ROC) curves was retrieved from logistic regression analysis and the area under the curve (AUC) was calculated. Univariate survival analysis was performed using the Kaplan-Meier method and the log-rank test. Multivariate analysis was conducted to determine an independent impact on survival using the Cox proportional hazard method. *P* < 0.05 was considered statistically significant.
